# Stress and stiffness as predictors of shear wave velocity in peripheral nerve

**DOI:** 10.1371/journal.pone.0319439

**Published:** 2025-03-11

**Authors:** Chelsea L. Rugel, Seth D. Thompson, Colin K. Franz, C. J. Heckman, Mitra Lavasani, Sabrina S. M. Lee

**Affiliations:** 1 Shirley Ryan AbilityLab, Chicago, Illinois, United States of America; 2 Department of Physical Medicine & Rehabilitation, Northwestern University Feinberg School of Medicine, Chicago, Illinois, United States of America; 3 Department of Physical Therapy and Human Movement Sciences, Northwestern University Feinberg School of Medicine, Chicago, Illinois, United States of America; 4 Department of Neurology, Northwestern University Feinberg School of Medicine, Chicago, Illinois, United States of America; 5 Department of Biomedical Physiology & Kinesiology, Simon Fraser University, Burnaby, British Columbia, Canada; Hamadan University of Medical Sciences, IRAN, ISLAMIC REPUBLIC OF

## Abstract

Shear wave elastography (SWE) is a promising non-invasive indicator for diagnosing peripheral neuropathy. Emerging validation studies using ultrasound-based measures of shear wave velocity (SWV) in other biological tissues, such as muscle, demonstrate there is a concern of whether SWE is an accurate measure of tensile stress or stiffness. Distinguishing between these two parameters and their relationship with SWV is crucial if SWE is to be used as a biomarker for peripheral neuropathies, where changes in mechanical properties are known to occur. In this study, we use cat sciatic nerves to first evaluate SWV in situ at knee positions known to reduce (90° flexion) or increase (180° extension) stress, and then excise nerves to directly quantify the relationships between SWV, stress, and stiffness with ex vivo tensile testing. Our ex vivo findings show that although SWV can be predicted using either stress or stiffness, stress explains more variability in sciatic nerve SWV. However, while stress remains the better predictor of SWV ex vivo, within the SWV range established in situ, stiffness improves its accuracy at estimating SWV, especially when also accounting for factors related to nerve viscoelasticity. Therefore, if SWE is to be used in clinical settings as an indicator of nerve stiffness in peripheral neuropathy, it is essential to standardize parameters such as limb positioning and nerve preloading, which could potentially mask pathological changes in nerve stiffness.

## Introduction

Peripheral neuropathy is a debilitating disorder that affects 1-3% of the general population, causing pain, weakness, and loss of sensation [1]. Standard clinical tests, including electrodiagnostic studies, are essential for assessing nerve functional impairment, but by the time functional deficits are detected structural damage can already be pervasive [2, 3]. Further understanding of nerve health is possible however by evaluating peripheral nerve mechanical properties, which are often altered by structural pathology [4–6]. Historically, nerve mechanical properties were only able to be studied ex vivo and required a patient biopsy to perform traditional tensile testing [5]. Such testing has revealed changes to nerve mechanical properties in the context of injury [7, 8], diabetes [9, 10], and exposure to chemotherapeutic agents [11]. However, obtaining nerve biopsies can cause further impairment [12], making tensile testing impractical.

Ultrasound shear wave elastography (SWE) eliminates the need for obtaining patient biopsies, as it is a noninvasive technique that can provide insight into mechanical properties by measuring shear wave velocity (SWV) through soft tissue [13]. Although SWE is relatively novel, it has been gaining momentum as a promising biomarker for detecting peripheral neuropathy [14–17]. Differences between impaired and unimpaired peripheral nerve SWV is frequently attributed to changes in stiffness due to the direct relationship between these properties in unstressed, isotropic, and linearly elastic materials [13]. Indeed, higher SWV has previously been observed in other pathological tissues associated with greater stiffness including liver fibrosis [18], breast cancer [19], and skeletal muscle contracture in patients with cerebral palsy [20]. However, biological tissues are constantly under mechanical stress, anisotropic, and nonlinearly elastic, which complicates the relationship between stiffness and SWV [13,15,21,22].

While there is evidence supporting the positive correlation between SWV and stiffness in passively stretched skeletal muscle [23–25], studies that decouple tensile stress from stiffness suggest that, under physiological loads, SWV is actually more dependent on tissue stress than stiffness [26, 27]. In peripheral nerves, the greatest source of nonpathological stress, elongation within the nerve bed during changes in limb position [28, 29], has previously been shown to significantly influence SWV [30–33]. However, to the best of our knowledge only one study has measured SWV while directly manipulating and recording force applied to the nerve [34], and none have applied uniaxial tensile force. Therefore, the relationship between peripheral nerve SWV, stress, and stiffness remains largely unknown. Characterizing this effect is nevertheless essential, as uniaxial tensile force is applied in parallel to the majority of nerve fibers in the nerve bed, and thus also to the propagation of most shear waves. Furthermore, this knowledge is crucial for using SWV to aid in the diagnosis of peripheral neuropathy, as electrodiagnostic studies and most clinical evaluations of nerve SWV are performed in the longitudinal plane [15,17].

The goal of this study is to quantify the effects of stress and stiffness on sciatic nerve SWV during tensile testing and compare these findings to limb position-induced changes in SWV. By bridging the gap between traditional ex vivo mechanical testing and in situ SWE evaluation under physiologically relevant conditions, we aim to quantify and determine the relationships between stress, stiffness, and SWV in nerve tissue. This characterization of how different factors influence peripheral nerve SWV is critical to optimizing the framework for SWE testing, and thus improving the quality of peripheral neuropathy clinical diagnosis.

## Materials and methods

All animals were obtained from a designated breeding establishment for scientific research and housed at Northwestern University’s Center for Comparative Medicine, an Association for Assessment and Accreditation of Laboratory Animal Care International (AAALAC International) accredited animal research program. All experimental procedures involving animals were reviewed and approved by the Institutional Animal Care and Use Committee (IACUC) at Northwestern University under approved protocol #IS00011448.

### Animals

Data presented here were obtained from five post-mortem male cats (*Felis catus*) weighing 4.73 ±  0.10 kg. Euthanasia was performed using a 2 mM/kg solution of KCl in addition to bilateral thoracotomy. Sciatic nerves from three individual cats were used for in situ experiments and sciatic nerves from two different cats were harvested for ex vivo testing. Cats were chosen as an animal model since their sciatic nerve thickness is substantially greater than the 1 mm spatial resolution of SWE for our clinical ultrasound machine [13].

### Ultrasound shear wave elastography

**Fig 1:**
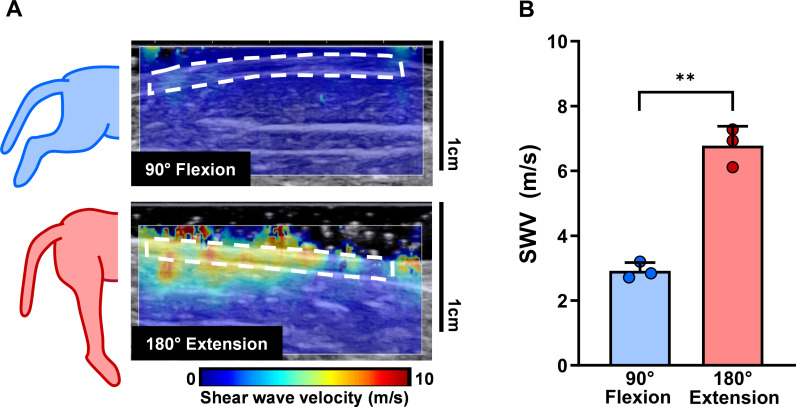
Sciatic nerve shear wave velocity (SWV) varies with limb position. (A) Representative B-mode ultrasound images with a shear wave elastogram overlay at knee positions associated with low (90° flexion) and high (180° extension) nerve stress. Analyzed nerve region of interest outlined by white dashed boxes. (B) Average sciatic nerve SWV across cats was significantly different between 90° knee flexion and 180° knee extension. Individual datapoints represent average SWV from five consecutively captured elastograms. Error bars indicate ±  SD. **p <  0.01 as determined by a two-tailed paired Student’s *t*-*t*est.

**Fig 2:**
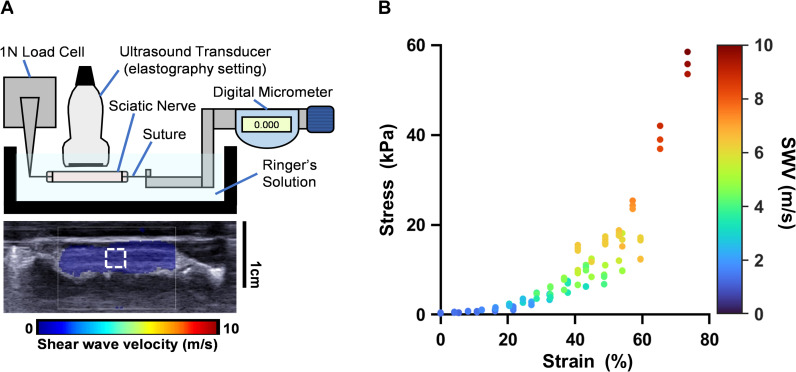
Tensile testing and simultaneous shear wave elastography (SWE) applied to ex vivo sciatic nerve. (A) Schematic of our tensile testing setup (not to scale) and representative B-mode ultrasound image of the sciatic nerve with shear wave elastogram overlay. White dashed box represents analyzed nerve region of interest. (B) Plotted are the stress-strain curves with corresponding SWV across both cats and relaxation times. Stress and SWV were captured simultaneously with three consecutive measurements after each elongation step.

An Aixplorer V9.1.1 ultrasonography system (SuperSonic Imagine, Aix-en-Provence, France) coupled with a 15MHz linear transducer array (256 elements, SuperLinear SL15–4, Vermon, Tours, France) in MSK Foot-Ankle mode was used for measuring SWV. For all experiments the ultrasound transducer was oriented parallel to the sciatic nerve in the longitudinal plane to facilitate shear wave propagation in the primary direction of nerve fiber alignment. SWE was applied continuously within a rectangular area that included the nerve. An elastogram, a colorimetric image containing a matrix of all SWV values at a single moment, and a standard B-mode ultrasound image were captured simultaneously (Fig 1A, Fig 2A) using a foot pedal trigger. All elastograms were processed and analyzed using custom software written in MATLAB R2021b (The MathWorks Inc., Natick, Massachusetts USA). For each elastogram, a binary mask was created to include a region of interest (ROI) of the sciatic nerve, including epineurial boundaries (Fig 1A, Fig 2A). SWV was calculated by averaging all elastography values within the binary mask. Additional details of this procedure have been previously described [35].

### In situ shear wave velocity

Immediately following euthanasia, cats (n =  3) were placed on their sides on a flat surface with the hip maintained at 90° flexion. The sciatic nerve was exposed along the middle of the femur and surrounded by ultrasound gel to maintain hydration and allow for conduction of shear waves. SWE was applied within a 1.25 x 2.00 cm rectangular area while the knee was manipulated into positions associated with low (90° flexion) and high (180° extension) nerve stress (Fig 1A) [36]. Order of knee position was randomly determined, and five elastograms were captured for each position.

### Sciatic nerve harvesting and storage for ex vivo testing

Sciatic nerve biopsies (n =  2) from cats different than those tested in situ, were harvested at a central location between the hip and knee immediately following euthanasia. Nerve biopsies were laid straight on a piece of gauze, under no tension, and stored flat in a sealed bag at -20 °C until testing.

### Tensile testing experimental setup

Nerve biopsies were thawed in their sealed bag in a water bath for 30 minutes prior to testing and were then placed into a customized chamber filled with room temperature Ringer’s solution (Fig 2A). Each end of the sciatic nerve was sutured into a tensile testing apparatus, with one end tied to the metal arm of a digital micrometer (Newport MT-RS, Irvine, California USA), and the other to the fixed lever arm of a 1 N load cell (Aurora Scientific 300C, Aurora, Canada). The ultrasound transducer was held in place by a three-prong clamp attached to magnetic indicator base, with the head of the transducer submerged in the chamber of Ringer’s solution to allow for proper conduction of ultrasound waves. SWE was then applied to a 5 x 5 mm area, with care taken to evaluate only the central portion of the sciatic nerve in order to avoid potential artifacts from the sutures.

### Tensile testing and shear wave velocity

During testing, voltage output from the ultrasound machine and load cell were continuously recorded in a custom MATLAB program. Voltage output from the load cell was converted to force in Newtons using the calibration constant provided by instrumentation specifications. Initial nerve length was defined as the distance between sutures at 0 N load output, representing the nerve under slack, and was subsequently manually elongated in 1 mm increments. Immediately following each nerve elongation step (R0), a foot pedal trigger was used to save three consecutive elastograms. Tensile force was matched to each elastogram at the onset of the foot pedal trigger, indicated by a change in ultrasound voltage output from + 5 V to 0 V. In order to account for transient viscoelastic effects of the nerve, three additional elastograms with corresponding force outputs were taken after a one-minute relaxation period (R1). Tensile testing was performed twice for each sciatic nerve.

### Calculating mechanical parameters

To avoid potential bias regarding nerve biopsy size, we report our findings in terms of tensile strain (nerve elongation divided by initial nerve length) and uniaxial tensile stress (tensile force divided by nerve cross-sectional area). Nerve cross-sectional area was modelled as a circle, similar to other studies of nerve mechanical properties [37, 38], using the average diameter (3.0 ±  0.1 mm under slack) from the continuous nerve boundaries of each ROI in the longitudinal plane. Since tensile testing was performed within nonlinear regions of the stress-strain curve (Fig 2B), tangent modulus, calculated as stress divided by strain at each elongation step, was used as a measure of nerve stiffness [34,39].

### Statistical analysis

**Fig 3:**
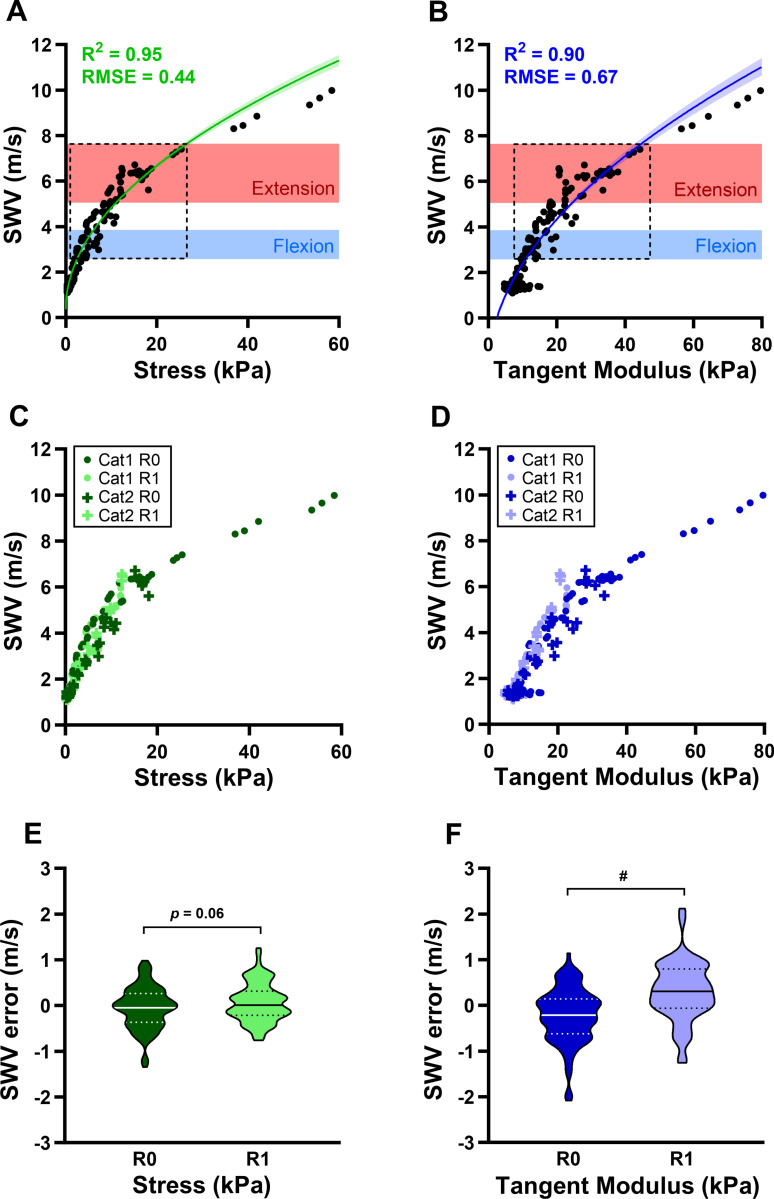
Ex vivo sciatic nerve shear wave velocity (SWV) is nonlinearly related to both stress and tangent modulus and is influenced by relaxation time. Nonlinear regression curves across all cats and relaxation times for (A) stress and SWV (R^2^ =  0.95, RMSE =  0.44) and (B) tangent modulus and SWV (R^2^ =  0.90, RMSE =  0.67). Transparent curve regions represent 95% confidence intervals. In situ sciatic nerve SWV range (horizontal rectangles) obtained during 90° knee flexion (blue) and 180° knee extension (red) are superimposed to contextualize data in respect to physiological conditions. Ex vivo SWV datapoints that fall within the in situ SWV range are shown inside dashed boxes. (C-D) Raw data broken down by cat (Cat1, Cat2) and relaxation time (R0: immediately after elongation, R1: one-minute after elongation). (E-F) Violin plot of SWV error from stress and tangent modulus nonlinear regression curves, grouped by relaxation time (R0, R1). Solid and dotted lines represent the median and interquartile range of SWV error, respectively. RMSE =  root mean square error. #p <  0.001 as determined by a two-tailed unpaired Student’s *t*-*t*est.

Statistical analyses were conducted in GraphPad Prism 10 (GraphPad Software, Boston, Massachusetts USA) and R (version 4.1.2) with an alpha level of 0.05. All results are presented as mean ±  standard deviation (SD), except for Fig 3E-F which are reported with standard error of the mean (SEM). The effect of knee position on in situ sciatic nerve SWV was evaluated using a two-tailed paired Student’s t-test.

To discern the nature of the relationships between SWV and stress and SWV and tangent modulus from ex vivo tensile testing, nonlinear regression analyses were conducted. Using the coefficient of determination (R^2^) and root mean square error (RMSE) to assess goodness of fit for each nonlinear regression, we determined that both stress and tangent modulus had a square root relationship with SWV. The effect of relaxation time on SWV error from both nonlinear regressions was assessed with two-tailed unpaired Student’s t-tests.

After applying a square root transformation to stress and tangent modulus, linear mixed-effects models (with cat included as a random factor) were used to elucidate whether stress or tangent modulus could best predict SWV. Normality of the residuals from all nonlinear models were evaluated with Kolmogorov-Smirnov tests. Likelihood ratio tests and AIC values were calculated to determine which linear model could best account for the variability in SWV. A Type III ANOVA with Satterthwaite’s method was used to ascertain which factors contributed most to each linear mixed-effects model.

## Results

To test if knee position, and thus stress indirectly, had an effect on sciatic nerve SWV, ultrasound SWE was performed on exposed cat nerves at 90° flexion and 180° extension (Fig 1A). Quantification of in situ sciatic nerve SWV revealed that at 180° knee extension, a position known to increase nerve stress, SWV was 132.21% greater (6.78 ±  0.60 m/s; **p <  0.01) than at 90° knee flexion (2.92 ±  0.25 m/s), a position that reduces stress on the nerve (Fig 1B).

In order to directly investigate the effects of stress and stiffness on nerve SWV ex vivo, cat sciatic nerve biopsies were tested in our customized apparatus (Fig 2A). The resulting stress-strain curves verified that measurements were not within the linear elastic range (Fig 2B), and thus our use of tangent modulus was indeed more appropriate than elastic modulus for estimating sciatic nerve stiffness. Our results also demonstrated that ex vivo sciatic nerve SWV fell within the range of in situ SWV, corresponding to stresses of 1.95 kPa −  25.36 kPa (Fig 3A) and tangent moduli of 9.65 kPa −  44.38 kPa (Fig 3B).

Both stress and tangent modulus exhibited a square root relationship with SWV, as determined by comparing RMSE and R^2^ values of multiple non-linear regressions. However, the nonlinear regression for stress (σ, in kilopascals) and SWV (*v* *,* in meters per second) demonstrated a better fit (R^2^ =  0.95, RMSE =  0.44; Eq 1, Fig 3A) than the regression for tangent modulus (E_t_, in kilopascals) and SWV (*v,* in meters per second) (R^2^ =  0.90, RMSE =  0.67; Eq 2, Fig 3B). Additionally, we observed that relaxation time was not a source of variability in the nonlinear regression for SWV and stress (p =  0.06, Figs 3C and 3E), but that it did contribute to the variability in the nonlinear regression for SWV and tangent modulus (Fig 3D) that contributed to SWV error (#p <  0.001, Fig 3F). Although this finding is not unexpected, as strain is used to calculate tangent modulus and held constant across relaxation times, it does emphasize how nerve viscoelasticity could affect SWV measurements.


$$v=\sqrt{\text{2}.00\sigma }\text{+ 0}.36$$ (1)

$$v=\sqrt{\text{2}.24E_{t}}-\text{2}.36$$ (2)

**Fig 4:**
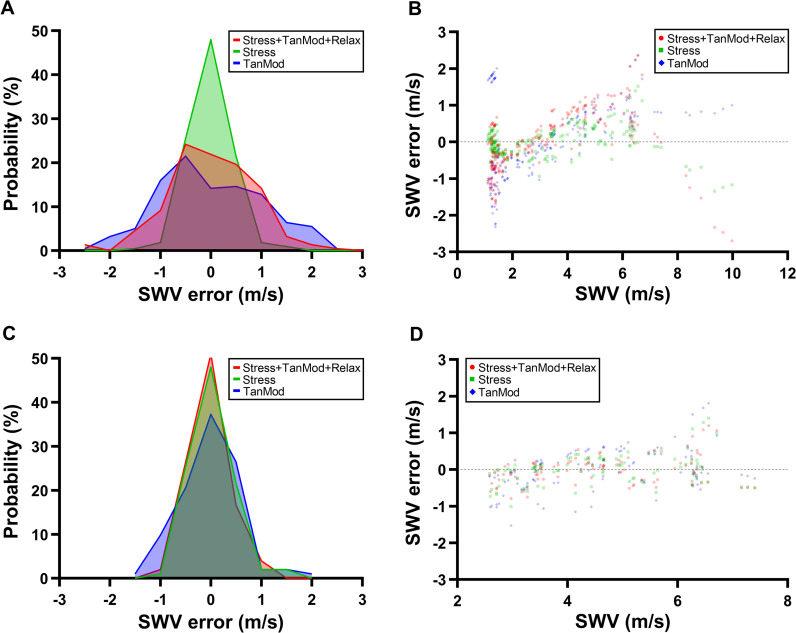
Ex vivo sciatic nerve shear wave velocity (SWV) error associated with linear mixed-effects models accounting for stress, tangent modulus, and relaxation time. (A and C) SWV error probability distribution and (B and D) SWV error comparison to measured SWV values from linear mixed-effects models. C and D represent linear mixed-effects models of ex vivo SWV values within in situ range (2.57-7.65 m/s). TanMod =  tangent modulus.

When stress, tangent modulus, and relaxation time are all accounted for, SWV is predicted more accurately (AIC =  241.62) than when stress (AIC =  242.12) or tangent modulus (AIC =  644.72) is used to determine SWV individually. However, in the multivariate model, stress explained significantly more of the variability in SWV (*F* =  1248.90, #p <  0.001) than tangent modulus (*F* =  5.08, * p <  0.05) or relaxation time (*F* =  7.16, **p <  0.01) as determined by a Type III ANOVA. Furthermore, likelihood ratio testing revealed that using only stress to model SWV was not significantly different than with tangent modulus and relaxation time also included (χ^2^ =  4.51, p =  0.10). Modeling SWV using only tangent modulus however was less accurate than when the model also accounted for stress and relaxation time (χ^2^ =  407.1, p <  0.001). Although the distribution of SWV errors from all three linear mixed-effects models passed Kolmogorov-Smirnov tests of normality (p >  0.05, Fig 4A), by plotting SWV error against measured SWV (Fig 4B), it is apparent that a large source of error from the tangent modulus model arises from SWV values below 2.57 m/s, the lowest in situ SWV measured. Additionally, many of the errors from all three models occurred at SWV values greater than 7.65 m/s, the highest in situ SWV measured.

To evaluate the ability of stress and tangent modulus to predict SWV under relevant physiological conditions, we re-ran each linear mixed-effects model using only measured SWV values within the in situ range of 2.57-7.65 m/s. The multivariate model accounting for stress, tangent modulus, and relaxation time remained superior (AIC =  122.23) compared to the individual stress (AIC =  126.62) and tangent modulus (AIC =  192.56) models. Interestingly, in the multivariate model stress explains less variability in SWV (*F* =  59.95, #p <  0.001) than in the multivariate model with the full range of SWV values, but is the only significant contributing factor as tangent modulus (*F* =  3.70, p =  0.06) and relaxation time (*F* =  0.47, p =  0.49) are no longer significant. However, the error in all in situ SWV range models is less than their full SWV range counterparts (Figs 4C and 4D), and when relaxation time and tangent modulus are used to model SWV without stress, the model improves (AIC =  163.38) compared to the tangent modulus only model (AIC =  192.56). Overall, these results suggest that under physiological conditions, not only is SWV more accurately predicted by stress, but that stiffness could be a better predictor of SWV when viscoelastic effects are accounted for.

## Discussion

This study provides much needed and novel insight into the relationship between SWE and peripheral nerve mechanical parameters by quantifying the direct relationships between stress, stiffness (tangent modulus), and SWV ex vivo. Additionally, all of our SWV measurements were made in the longitudinal plane, in parallel with the majority of nerve fibers and tensile force being applied. This allowed us to compare our ex vivo findings to those from our in situ experiments, which mirrored more clinically relevant conditions as the nerve was intact within the nerve bed.

Under physiological conditions in situ, knee positioning associated with higher stress (180° extension) [36] generated significantly greater nerve SWV than knee positioning associated with lower stress (90° flexion), analogous to prior in vivo human studies [30,32]. This finding of greater SWV at joint angles that elongate the nerve within the nerve bed has also been observed in upper extremity nerves [30, 31]. However, the effect of limb position and measurement location along different nerve paths warrants additional study, as both nerve tension [5,40] and SWV [30,32] are known to vary depending on number of joints in the limb and proximity to joints and other compression sites.

Unlike in situ, our ex vivo tensile testing of the sciatic nerve allowed us to directly manipulate and measure nerve stress. This tensile testing yielded a stress-strain curve similar to previously published reports at low stress levels [34,37,38] and contained SWV values within the in situ SWV range from both knee positions. SWV could be most accurately predicted using a linear mixed-effects model, accounting for stress, stiffness, and relaxation time. All three factors contributed significantly to the model, but stress explained most of the variability in SWV, a finding akin to studies in passively stretched skeletal muscle [26]. For ex vivo SWV values within the in situ SWV range, stress was the only significant contributing factor to the model and could similarly predict SWV without stiffness and relaxation time included as covariates. However, when stiffness was used to model SWV without stress, the accuracy of SWV predictions greatly improved by adding relaxation time as a covariate, highlighting the importance of considering viscoelastic factors when using SWV as an indicator of nerve stiffness.

SWV is frequently used to estimate material stiffness due to the direct mathematical conversion between these factors in isotropic, unstressed, and linearly elastic materials [13]. However, biological tissues, including peripheral nerves, violate these material assumptions. Nerves are anisotropic due to their heterogenous geometry and composition of nonuniformly distributed fascicles, vasculature, and connective tissue surrounded by collagenous epineurium. Additionally, nerves are continuously under stress from the tether of their paraneural sheath to surrounding tissues, are nonlinearly elastic largely due to their compositional ratio of elastin to collagen, and have viscous elements such as myelin. This complicates the relationship between stiffness and SWV.

Therefore, quantification of the relationships between peripheral nerve SWV, stress, and stiffness, such as those described herein, are crucial to understanding what aspects of nerves SWE is measuring, and thus what pathological mechanisms ultrasound SWE can best detect. Peripheral nerve SWE has already been found to be a potential biomarker for diabetic [15, 16] and entrapment [14,37] neuropathies, with greater nerve SWV consistently observed in impaired nerves. However, the pathological reason for this increase in SWV is largely unknown. Hypotheses include structural changes such as basement membrane thickening [41] or nonenzymatic crosslinking of fibers within the extracellular matrix [42], as well as greater external forces applied to the nerve especially at sites prone to compression [43]. By elucidating the effects of stress and stiffness on SWV, we can better optimize parameters for clinical assessments based on the pathological mechanism of action being studied. For instance, if SWV is more influenced by stress than stiffness, it would be beneficial to reduce the effect of nerve tension through limb positioning [28,29,36], stretching [44, 45], and/or preloading [46] to better evaluate intrinsic mechanical properties of the nerve.

Our study provides critical insight into the ability of nerve stress and stiffness to predict SWV, but was limited by small sample size, use of only male cats, and the inability to evaluate the same nerve both in situ and ex vivo. In particular, the potential influence of sex differences on nerve stress, stiffness, and SWV, merits further investigation. Although previous studies suggest that there is no significant effect of sex on either peripheral nerve stiffness in rodent models [47, 48] or on SWV in humans [10,49], research into sex differences in larger animal models has thus far been fairly limited. In other tissues however, sex hormones such as estrogen have been shown to influence connective tissue synthesis and biomechanics [50, 51]. As connective tissue is a key structural component of peripheral nerves, additional research is needed into whether sex contributes to differences in nerve SWV and mechanical properties.

Our study was also limited by methodological constraints. Of note, we were unable to assess the frequency behavior of shear wave speeds due to proprietary constrictions of our clinical ultrasound machine, and our experimental setup precluded the automation of strain rate and measurement of changes in nerve diameter during elongation. Furthermore, although our cat sciatic nerve biopsies were larger than the 1 mm spatial resolution of our ultrasound’s shear wave elastography system [13], due to their small size, it is possible that some of the shear waves became guided waves at the interface of the epineurium. Using a high-resolution camera system and an ultrasound machine with accessible frequency behavior data to quantify the relationship between nerve thickness, geometry, and SWV would thus be greatly beneficial. Ideally, such experiments, as well as those involving sex differences, would be conducted on both healthy and pathologic human nerves in order to improve translatability and further our understanding of factors that influence SWV in the context of disease.

## Supporting information

S1 File Raw in situ data. (XLSX)

S2 File Raw ex vivo data. (XLSX)

## References

[1] Hanewinckel R, van Oijen M, Ikram MA, van Doorn PA. The epidemiology and risk factors of chronic polyneuropathy. Eur J Epidemiol. 2016;31(1):5–20. doi: 10.1007/s10654-015-0094-6 26700499 PMC4756033

[2] Mohseni S, Badii M, Kylhammar A, Thomsen NOB, Eriksson K-F, Malik RA, et al. Longitudinal study of neuropathy, microangiopathy, and autophagy in sural nerve: Implications for diabetic neuropathy. Brain Behav. 2017;7(8):e00763. doi: 10.1002/brb3.763 28828222 PMC5561322

[3] Herrmann DN, Ferguson ML, Pannoni V, Barbano RL, Stanton M, Logigian EL. Plantar nerve AP and skin biopsy in sensory neuropathies with normal routine conduction studies. Neurology. 2004;63(5):879–85. doi: 10.1212/01.wnl.0000137036.26601.84 15365140

[4] Akhtar R, Sherratt MJ, Cruickshank JK, Derby B. Characterizing the elastic properties of tissues. Mater Today (Kidlington). 2011;14(3):96–105. doi: 10.1016/S1369-7021(11)70059-1 22723736 PMC3378034

[5] Topp KS, Boyd BS. Structure and biomechanics of peripheral nerves: nerve responses to physical stresses and implications for physical therapist practice. Phys Ther. 2006;86(1):92–109. doi: 10.1093/ptj/86.1.92 16386065

[6] Handorf AM, Zhou Y, Halanski MA, Li W-J. Tissue stiffness dictates development, homeostasis, and disease progression. Organogenesis. 2015;11(1):1–15. doi: 10.1080/15476278.2015.1019687 25915734 PMC4594591

[7] Borschel GH, Kia KF, Kuzon WM Jr, Dennis RG. Mechanical properties of acellular peripheral nerve. J Surg Res. 2003;114(2):133–9. doi: 10.1016/s0022-4804(03)00255-5 14559438

[8] Beel JA, Groswald DE, Luttges MW. Alterations in the mechanical properties of peripheral nerve following crush injury. J Biomech. 1984;17(3):185–93. doi: 10.1016/0021-9290(84)90009-5 6736055

[9] Chen R-J, Lin C-CK, Ju M-S. In situ transverse elasticity and blood perfusion change of sciatic nerves in normal and diabetic rats. Clin Biomech (Bristol). 2010;25(5):409–14. doi: 10.1016/j.clinbiomech.2010.01.013 20172636

[10] Dikici AS, Ustabasioglu FE, Delil S, Nalbantoglu M, Korkmaz B, Bakan S, et al. Evaluation of the Tibial Nerve with Shear-Wave Elastography: A Potential Sonographic Method for the Diagnosis of Diabetic Peripheral Neuropathy. Radiology. 2017;282(2):494–501. doi: 10.1148/radiol.2016160135 27643671

[11] Bober BG, Shah SB. Paclitaxel alters sensory nerve biomechanical properties. J Biomech. 2015;48(13):3559–67. doi: 10.1016/j.jbiomech.2015.07.020 26321364

[12] Nathani D, Spies J, Barnett MH, Pollard J, Wang M-X, Sommer C, et al. Nerve biopsy: Current indications and decision tools. Muscle Nerve. 2021;64(2):125–39. doi: 10.1002/mus.27201 33629393 PMC8359441

[13] Bercoff J, Tanter M, Fink M. Supersonic shear imaging: a new technique for soft tissue elasticity mapping. IEEE Trans Ultrason Ferroelectr Freq Control. 2004;51(4):396–409. doi: 10.1109/tuffc.2004.1295425 15139541

[14] Lin C-P, Chen I-J, Chang K-V, Wu W-T, Özçakar L. Utility of Ultrasound Elastography in Evaluation of Carpal Tunnel Syndrome: A Systematic Review and Meta-analysis. Ultrasound Med Biol. 2019;45(11):2855–65. doi: 10.1016/j.ultrasmedbio.2019.07.409 31402226

[15] Wee TC, Simon NG. Ultrasound elastography for the evaluation of peripheral nerves: A systematic review. Muscle Nerve. 2019;60(5):501–12. doi: 10.1002/mus.26624 31269240

[16] Dong B, Lyu G, Yang X, Wang H, Chen Y. Shear wave elastography as a quantitative biomarker of diabetic peripheral neuropathy: A systematic review and meta-analysis. Front Public Health. 2022;10 915883. doi: 10.3389/fpubh.2022.915883 35937233 PMC9354049

[17] Kantarci F, Ustabasioglu FE, Delil S, Olgun DC, Korkmazer B, Dikici AS, et al. Median nerve stiffness measurement by shear wave elastography: a potential sonographic method in the diagnosis of carpal tunnel syndrome. Eur Radiol. 2014;24(2):434–40. doi: 10.1007/s00330-013-3023-7 24220753

[18] Herrmann E, de Lédinghen V, Cassinotto C, Chu WC-W, Leung VY-F, Ferraioli G, et al. Assessment of biopsy-proven liver fibrosis by two-dimensional shear wave elastography: An individual patient data-based meta-analysis. Hepatology. 2018;67(1):260–72. doi: 10.1002/hep.29179 28370257 PMC5765493

[19] Youk JH, Gweon HM, Son EJ. Shear-wave elastography in breast ultrasonography: the state of the art. Ultrasonography. 2017;36(4):300–9. doi: 10.14366/usg.17024 28513127 PMC5621798

[20] Lee SSM, Gaebler-Spira D, Zhang L-Q, Rymer WZ, Steele KM. Use of shear wave ultrasound elastography to quantify muscle properties in cerebral palsy. Clin Biomech (Bristol). 2016;31 20–8. doi: 10.1016/j.clinbiomech.2015.10.006 26490641 PMC4729598

[21] Sigrist RMS, Liau J, Kaffas AE, Chammas MC, Willmann JK. Ultrasound Elastography: Review of Techniques and Clinical Applications. Theranostics. 2017;7(5):1303–29. doi: 10.7150/thno.18650 28435467 PMC5399595

[22] Doherty JR, Trahey GE, Nightingale KR, Palmeri ML. Acoustic radiation force elasticity imaging in diagnostic ultrasound. IEEE Trans Ultrason Ferroelectr Freq Control. 2013;60(4):685–701. doi: 10.1109/TUFFC.2013.2617 23549529 PMC3679553

[23] Bernabei M, Lee SSM, Perreault EJ, Sandercock TG. Shear wave velocity is sensitive to changes in muscle stiffness that occur independently from changes in force. J Appl Physiol (1985). 2020;128(1):8–16. doi: 10.1152/japplphysiol.00112.2019 31556833 PMC6985815

[24] Eby SF, Song P, Chen S, Chen Q, Greenleaf JF, An K-N. Validation of shear wave elastography in skeletal muscle. J Biomech. 2013;46(14):2381–7. doi: 10.1016/j.jbiomech.2013.07.033 23953670 PMC3818126

[25] Maïsetti O, Hug F, Bouillard K, Nordez A. Characterization of passive elastic properties of the human medial gastrocnemius muscle belly using supersonic shear imaging. J Biomech. 2012;45(6):978–84. doi: 10.1016/j.jbiomech.2012.01.009 22326058

[26] Bernabei M, Lee SSM, Perreault EJ, Sandercock TG. Axial stress determines the velocity of shear wave propagation in passive but not active muscles in vivo. J Appl Physiol (1985). 2023;134(4):941–50. doi: 10.1152/japplphysiol.00125.2022 36861673 PMC10069958

[27] Martin JA, Brandon SCE, Keuler EM, Hermus JR, Ehlers AC, Segalman DJ, et al. Gauging force by tapping tendons. Nat Commun. 2018;9(1):1592. doi: 10.1038/s41467-018-03797-6 29686281 PMC5913259

[28] Kleinrensink GJ, Stoeckart R, Mulder PG, Hoek G, Broek T, Vleeming A, et al. Upper limb tension tests as tools in the diagnosis of nerve and plexus lesions. Anatomical and biomechanical aspects. Clin Biomech (Bristol). 2000;15(1):9–14. doi: 10.1016/s0268-0033(99)00042-x 10590339

[29] Ridehalgh C, Moore A, Hough A. Sciatic nerve excursion during a modified passive straight leg raise test in asymptomatic participants and participants with spinally referred leg pain. Man Ther. 2015;20(4):564–9. doi: 10.1016/j.math.2015.01.003 25650068

[30] Greening J, Dilley A. Posture-induced changes in peripheral nerve stiffness measured by ultrasound shear-wave elastography. Muscle Nerve. 2017;55(2):213–22. doi: 10.1002/mus.25245 27396239

[31] Rugel CL, Franz CK, Lee SSM. Influence of limb position on assessment of nerve mechanical properties by using shear wave ultrasound elastography. Muscle Nerve. 2020;61(5):616–22. doi: 10.1002/mus.26842 32086830

[32] Andrade RJ, Freitas SR, Hug F, Coppieters MW, Sierra-Silvestre E, Nordez A. Spatial variation in mechanical properties along the sciatic and tibial nerves: An ultrasound shear wave elastography study. J Biomech. 2022;136:111075. doi: 10.1016/j.jbiomech.2022.111075 35390647

[33] Zhu B, Yan F, He Y, Wang L, Xiang X, Tang Y, et al. Evaluation of the healthy median nerve elasticity: Feasibility and reliability of shear wave elastography. Medicine (Baltimore). 2018;97(43):e12956. doi: 10.1097/MD.0000000000012956 30412114 PMC6221628

[34] Schrier VJMM, Lin J, Gregory A, Thoreson AR, Alizad A, Amadio PC, et al. Shear wave elastography of the median nerve: A mechanical study. Muscle Nerve. 2020;61(6):826–33. doi: 10.1002/mus.26863 32170959

[35] Lee SSM, Spear S, Rymer WZ. Quantifying changes in material properties of stroke-impaired muscle. Clin Biomech (Bristol). 2015;30(3):269–75. doi: 10.1016/j.clinbiomech.2015.01.004 25638688 PMC7057856

[36] Ellis R, Rohan M, Fox J, Hitt J, Langevin H, Henry S. Ultrasound Elastographic Measurement of Sciatic Nerve Displacement and Shear Strain During Active and Passive Knee Extension. J Ultrasound Med. 2018;37(8):2091–103. doi: 10.1002/jum.14560 29430675

[37] Abrams RA, Butler JM, Bodine-Fowler S, Botte MJ. Tensile properties of the neurorrhaphy site in the rat sciatic nerve. J Hand Surg Am. 1998;23(3):465–70. doi: 10.1016/S0363-5023(05)80464-2 9620187

[38] Kwan MK, Wall EJ, Massie J, Garfin SR. Strain, stress and stretch of peripheral nerve. Rabbit experiments in vitro and in vivo. Acta Orthop Scand. 1992;63(3):267–72. doi: 10.3109/17453679209154780 1609588

[39] Giannessi E, Stornelli MR, Sergi PN. Strain stiffening of peripheral nerves subjected to longitudinal extensions in vitro. Med Eng Phys. 2020;76 47–55. doi: 10.1016/j.medengphy.2019.10.012 31882395

[40] Phillips JB, Smit X, De Zoysa N, Afoke A, Brown RA. Peripheral nerves in the rat exhibit localized heterogeneity of tensile properties during limb movement. J Physiol. 2004;557(Pt 3):879–87. doi: 10.1113/jphysiol.2004.061804 15064329 PMC1665165

[41] Giannini C, Dyck PJ. Basement membrane reduplication and pericyte degeneration precede development of diabetic polyneuropathy and are associated with its severity. Ann Neurol. 1995;37(4):498–504. doi: 10.1002/ana.410370412 7717686

[42] King RH. The role of glycation in the pathogenesis of diabetic polyneuropathy. Mol Pathol. 2001;54(6):400–8. 11724915 PMC1187130

[43] Wang Y, Qiang B, Zhang X, Greenleaf JF, An K-N, Amadio PC, et al. A non-invasive technique for estimating carpal tunnel pressure by measuring shear wave speed in tendon: a feasibility study. J Biomech. 2012;45(16):2927–30. doi: 10.1016/j.jbiomech.2012.09.002 23031416

[44] Andrade RJ, Freitas SR, Hug F, Le Sant G, Lacourpaille L, Gross R, et al. Chronic effects of muscle and nerve-directed stretching on tissue mechanics. J Appl Physiol (1985). 2020;129(5):1011–23. doi: 10.1152/japplphysiol.00239.2019 32853116

[45] Neto T, Freitas SR, Andrade RJ, Vaz JR, Mendes B, Firmino T, et al. Shear Wave Elastographic Investigation of the Immediate Effects of Slump Neurodynamics in People With Sciatica. J Ultrasound Med. 2020;39(4):675–81. doi: 10.1002/jum.15144 31633231

[46] Keir PJ, Rempel DM. Pathomechanics of peripheral nerve loading. Evidence in carpal tunnel syndrome. J Hand Ther. 2005;18(2):259–69. doi: 10.1197/j.jht.2005.02.001 15891983

[47] Gupta RS, Berrellez D, Chhugani N, Luna Lopez C, Maldonado A, Shah SB. Effects of paclitaxel on the viscoelastic properties of mouse sensory nerves. J Biomech. 2021;115:110125. doi: 10.1016/j.jbiomech.2020.110125 33257008

[48] Petit E, Bavykina V, Thibault M, Bilodeau A, Choinière W, Brosseau J-P, et al. Assessing tissue mechanical properties: Development of a custom-made tensile device and application on rodents sciatic nerves. J Mech Behav Biomed Mater. 2024;159:106709. doi: 10.1016/j.jmbbm.2024.106709 39216337

[49] Bedewi MA, Elsifey AA, Alfaifi T, Kotb MA, Abdelgawad MS, Bediwy AM, et al. Shear wave elastography of the tibial nerve in healthy subjects. Medicine (Baltimore). 2021;100(3):e23999. doi: 10.1097/MD.0000000000023999 33545992 PMC7837829

[50] Nallasamy S, Yoshida K, Akins M, Myers K, Iozzo R, Mahendroo M. Steroid Hormones Are Key Modulators of Tissue Mechanical Function via Regulation of Collagen and Elastic Fibers. Endocrinology. 2017;158(4):950–62. doi: 10.1210/en.2016-1930 28204185 PMC5460796

[51] Hansen M, Kongsgaard M, Holm L, Skovgaard D, Magnusson SP, Qvortrup K, et al. Effect of estrogen on tendon collagen synthesis, tendon structural characteristics, and biomechanical properties in postmenopausal women. J Appl Physiol (1985). 2009;106(4):1385–93. doi: 10.1152/japplphysiol.90935.2008 18927264

